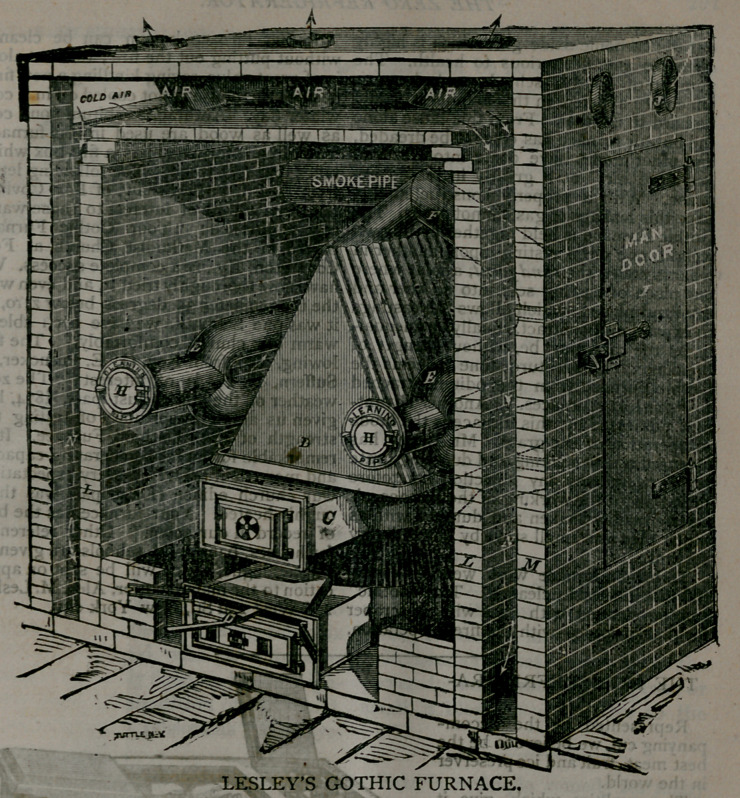# How Shall We Warm Our Houses?

**Published:** 1875-09

**Authors:** 


### HOW SHALL WE WARM OUR HOUSES?

If it should be asked what subject engages the most attention in our dwellings, schools and churches, the answer would at once be given, "Heating." The ingenuity of man has been taxed to the utmost to arrive at the point, of giving the greatest amount of heat with a given amount of coal. From the old Dutch plan, which consists of warming by means of earthen stoves—and which are still in use in the north of Europe—to the late invention of steam heaters and hot-air furnaces—people are still in doubt which is the best. It is not the purpose of this article to make comparisons, but to show to our readers the advantages obtained in the use of the "Gothic Furnace." It is absolutely essential in the construction of a furnace, or any heating apparatus, to so fasten the different parts that there can be no leakage of gas. In nearly all the present modes of construction, the different pieces of castings are held together by means of bolts; to this there is a decided objection. When two pieces of casting which are held together by such means are heated, it often results that one piece of casting will expand more or less than its counterpart, in consequence of which a breakage occurs at the bolt, letting out the carbonic oxide gas, which is highly offensive and deleterious to health. The number of houses, schools and churches which are afflicted in this manner it would be hard to number. From this cause alone the hot-air furnace has come to be dreaded, and other modes have come into use, "to get pure air" at a greatly increased expense. With a properly constructed furnace this leakage of gas cannot occur. The Gothic Furnace is held together by its own weight, one piece resting upon another in what is called the *sand joint*, a joint where one piece of casting sets into another and dry sand placed, which allows the castings to expand and contract, equally or unequally, as the case may be, without danger of breakage. This feature alone redeems the Gothic Furnace from the odium attached to "gas leaky furnaces." Another advantage possessed by this furnace is the mode of operation of the grate. Mr. Lesley has invented a new shaking and dumping grate which overcomes any objection heretofore raised about the working of the furnace; it can be readily shaken and dumped with as much ease as a small stove by any ordinary servant.

To have a furnace work well it is essential that it be easily cleaned. The "Gothic" can be cleaned with ease with a scraper for that purpose—with the fire in operation. No other furnace known can be cleaned without putting out the fire; this fact alone is of great value, saving kindling a new fire; besides the house is not cooled off in a cold winter day. Anthracite or bituminous coal as well as wood are used in this furnace. The furnace for wood has a fire-box which takes four feet wood. The following letter from Rev. J. P. Bradshaw, of Fort Covington, Ky., will be of interest to those wanting a furnace: "Your Gothic Furnace placed in the Methodist Church at Fort Covington, Ky., is a complete success. We have now thoroughly tried it, and even with the thermometer 35 degrees below zero, as it was one Sabbath, we have been able to warm our church comfortably." The following, also, from Rev. G. E. Purucker, of Suffern, N. Y., is of interest: "The zero weather of the winter just past, 1874, has given us ample opportunity of testing the strength of the 'Gothic' Furnace. It is remarkable for its heat-generating capacity and more than answered every expectation. Our church edifice (Episcopal) was thoroughly heated. Your furnace has the best of record in our town." Other references for houses, banks and schools are given in the catalogue, which will be sent on application to the manufacturer, Alex. M. Lesley, 226 West 23d St., New York city.

## Figures and Tables

**Figure f1:**